# Supplementary material for: A spatial interpolation method based on 3D-CNN for soil petroleum hydrocarbon pollution
Source: PLoS One. 2025 Jan 24;20(1):e0316940. doi: 10.1371/journal.pone.0316940 (PMC11759995; doi:10.1371/journal.pone.0316940)
Supplement: Attachment — Submitted filename: Response to reviewers.docx [file pone.0316940.s001.docx]

**Dear editor:**

Thanks for your and Reviewer’s valuable comments and suggestions for our manuscript entitled ***“A spatial interpolation method based on 3D-CNN for soil petroleum hydrocarbon pollution”****.*

We carefully read the comments of each reviewer and We have revised the manuscript carefully according to the advice raised by the Reviewers. Revised parts are highlighted with ‘Yellow’ colour in the manuscript. The detailed response to comments is listed point by point below:

***Review 1***

***Comment 1***

*If you are unable to adhere to our open data policy, please kindly revise your statement to explain your reasoning and we will seek the editor's input on an exemption. Please be assured that, once you have provided your new statement, the assessment of your exemption will not hold up the peer review process.*

***Response of Comment 1***

We will still maintain this option if it meets the requirements of the journal.

***Comment 2***

*In your Methods section, please provide additional information regarding the permits you obtained for the work. Please ensure you have included the full name of the authority that approved the field site access and, if no permits were required, a brief statement explaining why.*

***Response of Comment 2***

We totally agree with the reviewer. The design rule of the CBOW model is to predict the central word for surrounding words, and the setting of hyperparameters is related to the corpus database used. Section 3.2 added explanations for training hyperparameters.

We hope that the revision is acceptable and look forward to hearing from you soon.

Best regards,

Correspondence: Chao Liu

Email: liuchao@qut.edu.cn
